# Supplementary material for: MetaRibo-Seq measures translation in microbiomes
Source: Nat Commun. 2020 Jun 29;11:3268. doi: 10.1038/s41467-020-17081-z (PMC7324362; doi:10.1038/s41467-020-17081-z)
Supplement: Supplementary file 10 — Supplementary Data 7 [file 41467_2020_17081_MOESM10_ESM.zip › File2/Confidence_High_Taxonomy/222531_out.krona.html]

Javascript must be enabled to view this page.

members
magnitude
magnitudeUnassigned
count
unassigned
taxon
rank

222531\_out


SRS023914\_contig\_number\_27461
5
1

1
superkingdom
2

1
phylum
1239

1
class
186801

186802
1
order

1
family
216572

genus
1
459786

species
1
1945593

SRS098571\_contig\_number\_536


SRS013687\_contig\_number\_contig-100\_2128.47071SRS051031\_contig\_number\_contig-100\_7020.142173SRS053356\_contig\_number\_contig-100\_18550.187204
3
